# The relationship between plasma taurine levels in early pregnancy and later gestational diabetes mellitus risk in Chinese pregnant women

**DOI:** 10.1038/s41598-021-87178-y

**Published:** 2021-04-12

**Authors:** Peng Ju Liu, Yanping Liu, Liangkun Ma, Lihong Liu, Ting Hu, Zhuoling An, Ai Min Yao, Liang Yu Xia

**Affiliations:** 1 Department of Clinical Nutrition, Peking Union Medical College Hospital, China Academic Medical Science and Peking Union Medical College, Beijing, 100730 People’s Republic of China; 2 Department of Obstetrics and Gynecology, Peking Union Medical College Hospital, China Academic Medical Science and Peking Union Medical College, Beijing, 100730 People’s Republic of China; 3 Pharmacy Department of Beijing Chao-Yang Hospital, Capital Medical University, Beijing, 100020 People’s Republic of China; 4 Department of Gynaecology and Obstetrics, Shunyi Women’s and Children’s Hospital, Beijing, People’s Republic of China; 5 Department of Clinical Laboratory, Peking Union Medical College Hospital, Peking Union Medical College Hospital, China Academic Medical Science and Peking Union Medical College, Beijing, 100730 People’s Republic of China

**Keywords:** Biomarkers, Endocrinology, Health care

## Abstract

Taurine is a sulfur-containing amino acid that plays an important role in glucose homeostasis. However, it remains unknown whether the plasma concentration of taurine affects the risk of later gestational diabetes mellitus (GDM) development. We recruited 398 singleton-pregnancy women and followed up them during the course of pregnancy. We measured the plasma concentrations of taurine based on blood samples collected at nine-week gestation on average and obtained the data regarding both mothers and their infants from medical records. There was a significant increment in the mean value of HOMA-β across the tertiles of plasma taurine in multiparous women rather than in primiparous women. After adjustment for confounders, an increase of plasma taurine was nominally and significantly associated with a decrease risk of GDM; moreover, women with plasma taurine concentrations in the lowest tertile and in the second tertile had a higher risk of GDM than did those with plasma taurine in the top tertile in multiparous women other than primiparous women. Plasma taurine level seems to be associated with insulin secretion in early pregnancy and be more closely associated with β-cell function and the risk of GDM development in multiparas in comparison to primiparas.

## Introduction

Gestational diabetes mellitus (GDM), which is common during pregnancy, is defined as impaired glucose intolerance with onset or recognition during pregnancy^1,2^. The prevalence of GDM is highest in the Middle East and some North African countries, with a median of 15.2% (interquartile range 8.8–20.0%), followed by South-East Asia (median 15.0%; range 9.6–18.3%)^3^. Although the lack of consensus and consistency in the diagnostic criteria for GDM leads to a large difference in the prevalence of GDM across countries and regions^3^, it is estimated to be approximately 15% among pregnant women in mainland China^4^, using the International Association of Diabetes and Pregnancy Study Groups (IADPSG) diagnostic criteria for GDM. GDM has a number of adverse effects for pregnant women and their offspring. For women with GDM, they are more likely to have preeclampsia, cesarean deliveries, shoulder dystocia and are likely to have a higher rate of developing type 2 diabetes mellitus (T2DM) in the postpartum period than those without GDM^5,6^. Also, the offspring of women who had GDM previously are more likely to develop obesity and abnormal blood glycemia in later life^5,7,8^. An increased insulin resistance and β-cell defects are involved in the metabolic abnormalities underlying GDM, and these most likely exist in many women who are going to get pregnant^3^. However, those defects are difficult to detect in early stage of pregnancy, as they are almost entirely asymptomatic. Moreover, the metabolic factors including increased endogenous glucose production and decreased peripheral insulin sensitivity add additional stress on β-cells^3^. Therefore, early recognition of β-cell function may be helpful to early prediction of GDM risk.

Taurine, as a conditionally essential amino acid which is widely distributed in mammalian tissues including the brain and heart, retina, blood cells, large intestine and secretary structures^9^, accounts for up to 0.1% of the total weight of the human body^10^. Taurine is a byproduct of the sulphurous amino acids methionine and cysteine. Along with its antiapoptotic and antioxidant activity, taurine exerts a variety of important biological effects on osmotic regulation, membrane stability, and modulation of calcium signaling neurotransmission^11^. Not only that, taurine also plays an important role in glucose homeostasis and against diabetes and its complication^10^, through at least three pathways: (1) the advanced glycation end-product pathway, (2) PI3-kinase/AKT pathway, and (3) mitochondrial apoptosis pathway^12^. Findings from both animal and human studies have suggested that taurine may improve β-cell insulin secretion or modulate the effects of diabetes-associated genes on improvement of insulin sensitivity^13,14^, suggesting that taurine may have benefits for individuals with diabetes through its effect on insulin secretion or sensitivity.

Given the observed relationship between taurine and diabetes, we hypothesized that plasma taurine levels might be associated with the development of GDM through its effects on β-cell function. To the best of our knowledge, there is little study evaluating the relationship between plasma taurine levels and GDM risk. Therefore, we aimed to analyse preliminary whether maternal first-trimester plasma taurine levels affect GDM risk.

## Results

### Characteristics of primiparous as well as multiparous women at baseline

**Figure 1:**
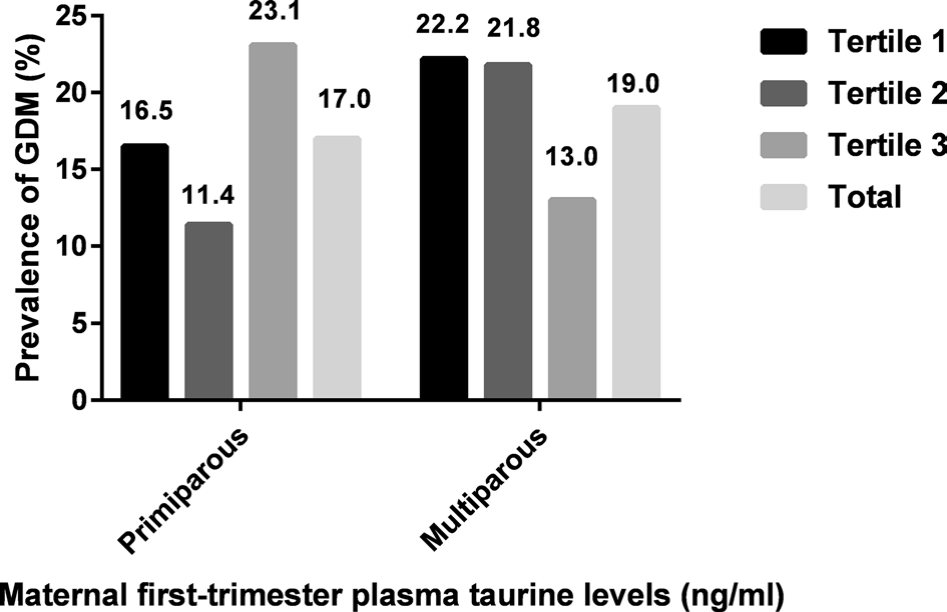
Prevalence of GDM at each tertile of taurine of both primiparous and multiparous women.

Of those 398 participants, 71 (17.8%) were diagnosed as having GDM; 40 of them were primiparas and the rest were multiparas. Compared with participants in primiparas group, women in multiparas group had a significantly higher age, BMI at enrolment, CRP level and FPG, but a lower ferritin level and a lower average education level (Table 1). The proportion of pregnant women diagnosed with GDM in each tertile of taurine of both primiparous and multiparous women is shown in Fig. 1, respectively. We did not report the participants’ habits of smoking and drinking, because no one has smoked or drunk for at least 3 months before recruitment.

**Table 1:** Baseline characteristics of study population.

Variables	All women (n = 398)	Primiparous (n = 235)	Multiparous (n = 163)	*P*
Age (years)	29.2 (3.6)	28.0 (3.2)	31.0 (3.6)	0.000
BMI at enrolment (kg/m^2^)	22.8 (3.8)	22.1 (3.5)	23.7 (4.0)	0.000
Weight gain (kg)^a^	9.1 (5.2)	9.1 (5.0)	9.2 (5.5)	0.565
Hemoglobin (g/L)	131.5 (10.3)	131.8 (10.7)	131.0 (9.8)	0.484
Ferritin (ng/ml)	51.0 (35.6)	54.7 (38.5)	45.7 (30.2)	0.01
Serum homocysteine (μmol/L)	9.0 (2.5)	9.0 (1.9)	9.1 (3.2)	0.529
C-reactive protein (mg/L)	3.0 (2.9)	2.4 (2.3)	3.8 (3.4)	0.000
HOMA-β	216.2 (158.0)	221.8 (171.3)	207.0 (135.3)	0.402
In tertile 1 of tautine		191.3 (140.4)	177.8 (138.6)	
In tertile 2 of tautine		225.9 (215.9)	187.9 (109.0)	
In tertile 3 of tautine		248.9 (147.4)	256.4 (145.0)	
HOMA-IR	1.86 (1.29)	1.77 (1.22)	1.98 (1.37)	0.126
Fasting plasma glucose (mmol/L)	4.5 (0.4)	4.4 (0.4)	4.5 (0.4)	0.01
Fasting insulin (mIU/ml)	9.2 (5.8)	8.8 (5.4)	9.7 (6.3)	0.129
Taurine (ng/ml)	657.6 (158.2)	662.2 (155.0)	656.9 (160.8)	0.377
History of PCOS	9 (2.3)	7 (3.0)	2 (1.2)	0.248
Family history of diabetes	36 (9.0)	18 (7.7)	18 (11.0)	0.287
Prevalence of GDM	71 (17.8)	40 (17.0)	31 (19.0)	0.690
In tertile 1 of tautine		13 (16.5)	12 (22.2)	
In tertile 2 of tautine		9 (11.4)	12 (21.8)	
In tertile 3 of tautine		18 (23.1)	7 (13.0)	
Oral multi-nutrients supplements	140 (35.2)	82 (34.9)	58 (35.6)	0.915
Physical activity				0.147
0–150 min per week	208 (52.3)	131 (55.7)	77 (47.2)	
≥ 150 min per week	190 (47.7)	104 (44.3)	86 (52.8)	
Education				0.000
Senior middle school or lower	83 (20.9)	34 (14.5)	49 (30.1)	
College degree or higher	315 (79.1)	201 (85.5)	114 (69.9)	

*BMI* body mass index, *HOMA-IR* homeostasis model assessment—insulin resistance, *HOMA-β* homeostasis model assessment-β, *PCOS* polycystic ovary syndrome, *GDM* gestational diabetes mellitus.

^a^Represents weight gain from enrolment to the 75-g oral glucose tolerance test.

In primiparous women, women in GDM group had high BMI, hemoglobin, HOMA-IR, FPG, and CRP values (all *P* < 0.05), and tended to be more likely to have family history of diabetes (*P* = 0.055) and history of PCOS (*P* = 0.065), compared with those without GDM; no other significant differences were observed between the two groups. Whereas in multiparous women, participants in GDM group were significantly older, had less exercise time per week (*P* = 0.011), and had higher FPG and HOMA-IR values (all *P* < 0.05); no other significant differences were found between the two groups (Table 2).

**Table 2:** Comparison of basic characteristics between GDM group and non-GDM group in primiparous and multiparous women.

Variables	Primiparous			Multiparous		
	Non-GDM (n = 195)	GDM (n = 40)	*P*	Non-GDM (n = 132)	GDM (n = 31)	*P*
Age (years)	27.9 (3.2)	28.5 (3.1)	0.314	30.5 (3.4)	33.2 (3.4)	0.000
BMI at enrolment (kg/m^2^)	21.6 (3.2)	24.3 (3.9)	0.000	23.5 (3.9)	24.8 (4.0)	0.096
Weight gain (kg)^a^	8.9 (5.4)	9.5 (4.4)	0.339	9.2 (5.7)	9.3 (4.5)	0.873
Hemoglobin (g/L)	131.0 (11.0)	135.6 (7.6)	0.013	131.4 (9.7)	129.5 (10.3)	0.336
Ferritin (ng/ml)	53.8 (36.3)	58.9 (48.4)	0.446	46.8 (31.4)	41.0 (24.6)	0.337
Serum homocysteine (μmol/L)	8.9 (1.9)	9.1 (2.1)	0.563	9.2 (3.5)	9.0 (1.3)	0.814
C-reactive protein (mg/L)	2.2 (2.1)	3.6 (2.9)	0.005	3.7 (3.5)	4.6 (3.1)	0.178
HOMA-β	220.4 (176.8)	228.1 (143.8)	0.797	209.5 (129.7)	196.5 (159.0)	0.632
HOMA-IR	1.59 (0.94)	2.68 (1.89)	0.001	1.81 (1.08)	2.68 (2.10)	0.032
Fasting plasma glucose (mmol/L)	4.4 (0.4)	4.7 (0.4)	0.000	4.5 (0.4)	4.9 (0.6)	0.000
Fasting insulin (mIU/ml)	8.1 (4.5)	12.2 (7.6)	0.000	9.1 (5.2)	12.4 (9.5)	0.009
Taurine (ng/ml)	655.5 (151.9)	694.8 (167.1)	0.145	662.6 (163.8)	632.7 (147.7)	0.352
History of PCOS	4 (2.1)	3 (7.5)	0.065	1 (0.8)	1 (3.2)	0.261
Family history of diabetes	12 (6.2)	6 (15.0)	0.055	13 (9.8)	5 (16.1)	0.315
Oral multi-nutrients supplements	71 (36.4)	11 (27.5)	0.281	50 (37.9)	8 (25.8)	0.202
Physical activity			0.196			0.011
0–150 min per week	105 (53.8)	26 (65.0)		56 (42.4)	21 (67.7)	
≥ 150 min per week	90 (46.2)	14 (35.0)		76 (57.6)	10 (32.3)	
Education			0.062			0.767
Senior middle school or lower	32 (16.4)	2 (5.0)		39 (29.5)	10 (32.3)	
College degree or higher	163 (83.6)	38 (95.0)		93 (70.5)	21 (67.7)	

*BMI* body mass index, *HOMA-IR* homeostasis model assessment—insulin resistance, *HOMA-β* homeostasis model assessment-β, *PCOS* polycystic ovary syndrome.

^a^Represents weight gain from enrolment to the 75-g oral glucose tolerance test.

### Association of plasma taurine concentrations with β-cell function

Partial correlation analyses showed that the plasma taurine level was positively related to the value of HOMA-β in both primiparas (r = 0.188, *P* = 0.004) and multiparas (r = 0.196, *P* = 0.015), with controlling for age, BMI, CRP, family history of diabetes, history of PCOS, and physical activity.

**Figure 2:**
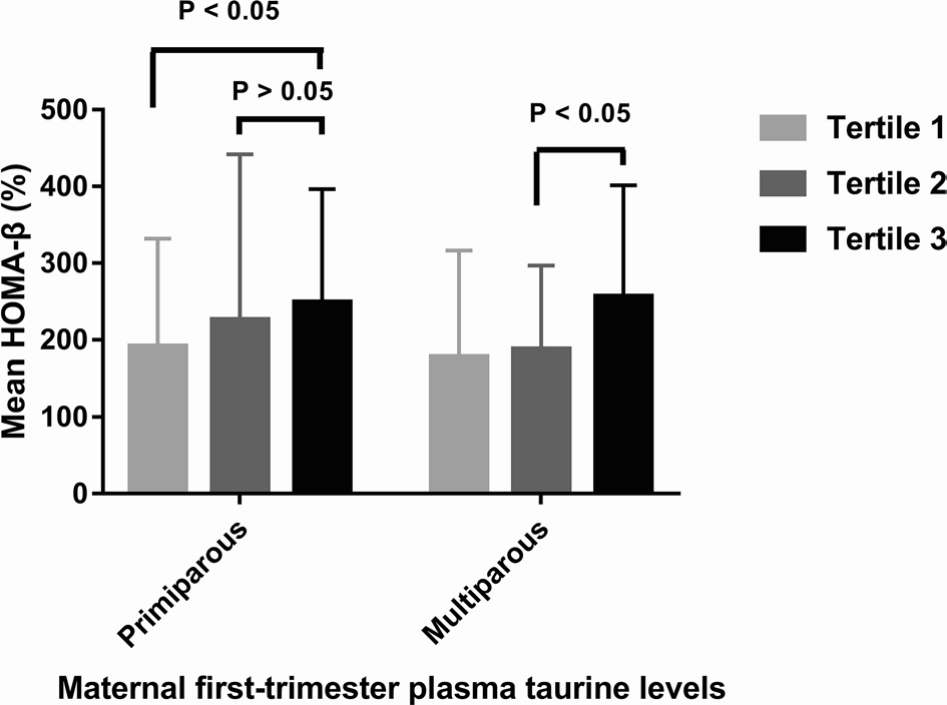
Mean HOMA-β value at each tertile of plasma taurine of both primiparous and multiparous women.

The mean HOMA-β value at each tertile of plasma taurine of both primiparous and multiparous women is shown in Fig. 2. In primiparous women, in general, the mean value of HOMA-β had a non-significant increasing trend (*P* > 0.05) across the tertiles of plasma taurine; but the mean value of HOMA-β in the first tertile was significantly lower than that in the third tertile. Whereas in multiparous women, there was a significant increasing trend (*P* < 0.05) in the mean value of HOMA-β across the tertiles of plasma taurine.

### Association of plasma taurine concentrations with later risk of GDM

Plasma taurine concentrations were divided into tertiles according to the cutoff points of the distribution for the primiparas and the multiparas, respectively. The top tertile was used as a reference.

In multiparous women, binary logistic analyses indicated that women with plasma taurine concentrations in the bottom (adjusted OR = 5.797, 95% CI = 1.398–24.031, *P* = 0.015) as well as in the middle tertile (adjusted OR = 4.739, 95% CI = 1.131–19.859, *P*  = 0.033) had a higher risk of GDM than did those with plasma taurine concentrations in the top tertile, respectively, after adjustments for the potential confounders described above (statistical analysis). When plasma taurine concentration was regarded as a continuous variable, the one-standard deviation (SD) increments of plasma taurine (160.8 ng/ml) was nominally and significantly associated with a decrease risk of GDM (adjusted OR = 0.997, 95% CI = 0.993–1.000, *P* = 0.041).

In primiparous women, binary logistic analyses revealed that women in the lower two tertiles of plasma taurine concentration did not have a significantly higher risk of GDM than those in the top tertile after adjustment for the same covariates as in the above regression model. Similarly, the plasma taurine concentrations were not significantly associated with GDM risk when plasma taurine concentration was regarded as a continuous variable (Table 3).

**Table 3:** Association of GDM risk with plasma taurine levels in primiparous and multiparous women.

Plasma taurine levels	GDM, n (%)	The risk of GDM^a^	
		Adjusted OR (95% CI)	*P*
**Primiparous**			
T1(< 594.2 ng/ml)	13 (16.5)	0.479 (0.174–1.322)	0.155
T2 (594.2–702.7 ng/ml)	9 (11.4)	0.941 (0.365–2.424)	0.899
T3 (> 702.7 ng/ml)	18 (23.1)	1.00 (Reference)	–
As a continuous variable (SD, 155.0 ng/ml)	–	1.001 (0.998–1.004)	0.512
**Multiparous**			
T1(< 579.9 ng/ml)	12 (22.2)	5.797 (1.398–24.031)	0.015
T2 (579.9–719.8 ng/ml)	12 (21.8)	4.739 (1.131–19.859)	0.033
T3 (> 719.8 ng/ml)	7 (13.0)	1.00 (Reference)	–
As a continuous variable (SD, 160.8 ng/ml)	–	0.997 (0.993–1.000)	0.041

^a^Adjusted for age, physical activity, education level, BMI, weight gain (from enrolment to the 75-g OGTT), family history of diabetes, history of PCOS, use of multi-nutrients supplements, C-reactive protein, hemoglobin, ferritin, and serum homocysteine.

## Discussion

The present study firstly reported that a higher level of maternal plasma taurine in the first trimester was significantly associated with an increased insulin secretion evaluated by HOMA-β index and with a lower GDM risk in multiparas other than in primiparas, suggesting that maternal plasma taurine in early pregnancy may be a fair indicator of secretion function of β-cells and also a potential predictor of GDM development in multiparas, not primiparas. However, the reasons for the discriminatory effects of plasma taurine on insulin secretion and GDM risk between primiparas and multiparas are unknown.

Taurine, as a conditionally essential amino acid, is widely distributed in tissues of animals and humans^9,15,16^. The beneficial effects of taurine against diabetes and its related complications have been widely reviewed in human clinical and animal studies^10,17^. On the one hand, it has been demonstrated that taurine deficiency can lead to various tissue dysfunction which may be the cause of diabetes complications of related tissue^17^; plasma taurine concentrations were significantly lower in individuals with diabetes in comparison to controls^18^. On the other hand, taurine exhibits hypoglycemic effect by improving insulin sensitivity, stimulating insulin secretion and reducing inflammation and oxidative stress^10^. Several possible mechanisms may be involved in taurine’s effects on maintaining glucose homeostasis. These possible mechanisms include as follows: taurine may prevent glucagon hypersecretion by modulating several pancreatic cells^19^; taurine may stimulate insulin secretion through inhibition of ATP sensitive K + channels^20^; taurine may improve insulin sensitivity by means of increasing the levels of insulin receptor substrate (IRS)-1 and 2 tyrosine and AKT serine phosphorylation^21^; taurine may suppress inflammatory factors as well as nuclear factor kappa-B (NF-κB) activity to decrease inflammation mediated destruction of pancreatic β cells^22^.

Although many experimental studies have been focused in the relationship between taurine and daibates mellitus, very few studies specifically examining the relationship between plasma taurine levels and GDM have been reported.

A number of GDM risk factors, such as advanced maternal age, ethnicity, parity, family history of T2DM, PCOS, and physically inactive lifestyle before and during pregnancy, have been identified by epidemiological studies^3^. To be noted, insulin resistance plays an important role in the pathophysiology of GDM. It should be noted that decreased peripheral insulin sensitivity before pregnancy has been found in women with normal glycemia before pregnancy but go on to develop GDM in late gestation^23^. Due to the adaptations of pancreatic β-cells, which increase the insulin response, these women initially adaptively maintain normal glycemia in early pregnancy. However, the insulin resistance increases along with the advanced pregnancy, when the capacity of insulin production and secretion is overwhelmed by rising insulin resistance, maternal hyperglycemia ensues^5^, not to mention that many pregnant women have existed insulin resistance and β-cell defects before conception^24^. Moreover, the function of β-cells seems to be different between primiparas and multiparas^25^. Unfortunately, it is still unknown whether plasma taurine levels affect insulin secretion or β-cell function of pregnant women or whether plasma taurine levels are associated with the risk of GDM development. In a small study, it was reported that maternal plasma taurine, measured at delivery, did not significantly differ in women with GDM compared to controls^26^. However, another small study reported that plasma taurine (measured after a median period of 6 years from index pregnancy) was significantly lower in women who had experienced GDM and was inversely related to the previous gestational area-under-curve glucose and had a positive relation to post-gestational C-peptide/FPG as well as to C-peptide/FPG measured during pregnancy^27^, suggesting that plasma taurine may be inversely related to insulin secretion during pregnancy, as well to that of women in postpartum. In the present study, we found that maternal first-trimester plasma taurine, measured at a mean gestation age of 9 weeks, was positively related to HOMA-β values in both multiparas and primiparas independent of age, BMI, CRP, family history of diabetes, history of PCOS, and physical activity. Furthermore, there was a significant increment in the mean value of HOMA-β (*P* < 0.05) across the tertiles of plasma taurine in multiparas rather than in primiparas. These results suggest that plasma taurine level of multiparas may be more closely associated with insulin secretion compared to that in primiparas. The specific reasons for the differences need to be further investigated.

In addition, the percentage of women who developed GDM later in each tertile of plasma taurine was also different between primiparas and multiparas in this study. In multiparas, the lowest rate of developing GDM occurred among the women with the top tertile of plasma taurine, which was contrary to that of the primipara (Fig. 1). Consistence with that, binary regression analyses showed that a higher plasma taurine level is an independent protective factor for the development of GDM in multiparas other than in primiparas. Moreover, binary logistic regression analyses showed that the risk of GDM increases in multiparas as plasma taurine levels decrease. One possible explanation for these results may be due to the different association of plasma taurine with HOMA-β between primiparas and multiparas.

As known, the binding of insulin to the cell surface insulin receptor in peripheral (such as skeletal muscle) results in glucose uptake by cells and subsequently leads to autophosphorylation by the tyrosine kinase domain of the insulin receptor β-subunit (IR-β). This process enables glucose uptake by peripheral cells^3^. It has been reported that the autophosphorylation of IR-β is lower in women with GDM than in controls^28^. In addition, the content of IRS1, one of the signaling molecular of insulin signaling cascade, is lower in skeletal muscle of pregnant women than that in non-pregnant women as the pregnancy advanced^3^. These states result in a lower glucose uptake. However, taurine may increase the levels of IRS-1/2 tyrosine and AKT serine phosphorylation^21^, which results in the improvement of insulin sensitivity and an increase of glucose uptake by cells. This may be another explanation for the association between plasma taurine and GDM. However, further studies are needed to explore why there is a different association of plasma taurine with GDM risk between primiparas and multiparas.

## Conclusion

The strengths of this study were as follows: (1) it is the first cohort study that investigates the association of maternal plasma taurine levels with GDM risk, (2) the researchers who collected the data of obstetric outcomes were blinded to plasma taurine concentrations. However, our study has several limitations. First, the concentrations of plasma taurine measured in the participants of this study were generally lower than those reported in other studies^27^, one possible explanation is due to the different methods of measurement; it is difficult to exclude the effect of matrix on the concentration of taurine. Second, any marker associated with the potential mechanism underlying between plasma taurine and GDM, including inflammatory factors, has not been measured in this study, thus this study was unable to provide an explanation of the relevant mechanism. Third, dietary taurine intake in the study population was not quantified. Fourth, only the plasma taurine concentrations in early pregnancy were measured in this study; for financial reasons, the plasma taurine concentrations in the second trimester of pregnancy were not measured, thus whether there is a change in plasma taurine concentration from the first to the second trimester of pregnancy were unknown. Finally, the sample size of this study is relatively small, and studies with larger sample sizes are warranted.

Despite the limitations, the present study, for the first time, found that a lower plasma taurine level in early pregnancy seems to be a fair marker of an inadequate insulin secretion and to be more closely associated with a higher risk of GDM development in multiparas in comparison to primiparas.

## Methods

This study protocol got approval of the Ethics Committee of Peking Union Medical College Hospital (Number of the approval document: hs-1646). In addition, this study was also registered on www.ClinicalTrials.gov (Registration ID: NCT03651934). The present study followed the Declaration of Helsinki. We have got the written informed consent from all participants before the recruitment.

### Participants

The participants in this study were from one cohort study which was conducted at the Shunyi Women’s and children’s Hospital of Beijing Children’s Hospital, Beijing, PR China, and they were recruited from October to December 2018. The detailed descriptions of the participants of the cohort can be found in our recent publications^29–31^.

We only included women who are singleton pregnancy and are the Han ethnicity. The exclusion criteria were well described elsewhere^31^. In addition, women with incomplete plasma taurine records were also excluded in the present study.

**Figure 3:**
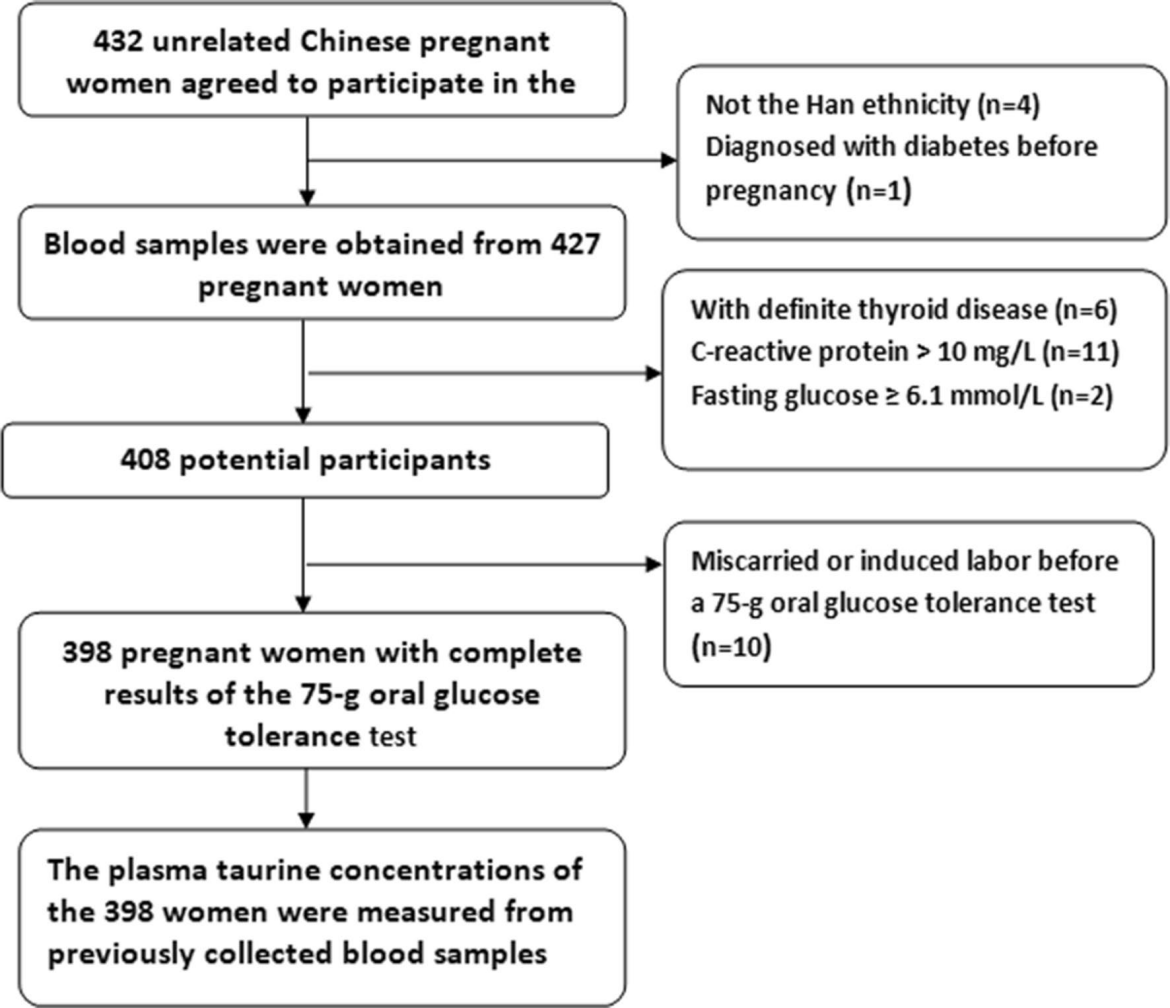
Participant flowchart.

Clinicodemographic information including age, education level, lifestyle factors (smoking habits, drinking habits, and physical activity), medical and family history, parity status, use of multi-nutrients supplements were collected by trained researchers through a standard questionnaire. Each participant was followed up in the same hospital during the course of pregnancy. A 75-g OGTT was routinely administered to all participants at 24–28 weeks’ gestation. Diagnosis of GDM was based on the IADPSG criteria^32^. Finally, a total of 398 eligible women were enrolled in this study. The participant flowchart is presented in Fig. 3.

### Anthropometric and blood sample measurements

Anthropometric measurements of the participants were well described in our published papers^29–31^. Blood samples were retrieved from all participants in the morning after an overnight (≥ 8 h) fast at their first visit (before 12 weeks’ gestation). We measured concentrations of hemoglobin, ferritin, homocysteine, FPG, insulin, and CRP, and the specific determination methods were described in our recent publications^29–31^. We also calculated homeostasis model assessment-β (HOMA-β) and homeostasis model assessment-insulin resistance (HOMA-IR). The calculation of HOMA-β and HOMA-IR was followed by the method of Matthews et al.^33^.

#### Measurement of plasma taurine concentrations

Plasma taurine concentrations were measured using fasting plasma samples that had been stored at –80℃ since collection. The samples were prepared and analysed by the Pharmacy Department of Beijing Chao-Yang Hospital, Capital Medical University, Beijing, China, by using the metabolomics analysis. Protein precipitation method was employed for the biological sample pretreatment. The detailed procedures are published elsewhere^34^.

### Statistical analysis

We performed the statistical analysis by using the SPSS (version 16.0, Chicago, IL, USA). Variables were described as mean (SD) or frequency (percentage), when appropriate. Data on primiparous and multiparous women were analysed separately, because insulin secretion evaluated by HOMA indices is lower in multiparas than in primiparas^25^. The independent-sample *t* test was used to compare continuous variables between women with and without GDM, whereas the chi-square test was used to examine categorical variables. Plasma taurine concentrations were divided into parity-specific tertiles (for primiparous women, T1, < 594.2 ng/ml; T2, 594.2–702.7 ng/ml; T3, > 702.7 ng/ml; whereas for multiparous women, T1, < 579.9 ng/ml; T2, 579.9–719.8 ng/ml; T3, > 719.8 ng/ml). The difference in HOMA-β values across the tertiles of plasma taurine in both primiparas and multiparas was investigated with univariate analysis of variance with adjustment for age, BMI (at enrolment), CRP, physical activity, family history of diabetes (yes, no), history of polycystic ovarian syndrome (PCOS) (yes, no), and use of multi-nutrients supplements (yes, no). Binary logistic regression analyses were used to estimate the odds ratios (ORs) and 95% confidence intervals (CIs) of the associations of GDM with plasma taurine levels (as tertiles or a continuous variable) in both primiparous and multiparous women, with adjustments for potential confounders, including age, physical activity, education level (high, low), BMI (at enrolment), weight gain (from enrolment to the 75-g OGTT), family history of diabetes, history of PCOS, use of multi-nutrients supplements, CRP, hemoglobin, ferritin, and serum homocysteine. Results with *P* < 0.05 were regarded statistically significant.

## Abbreviations

GDM: Gestational diabetes mellitus
FPG: Fasting plasma glucose
IGT: Impaired glucose tolerance
T2DM: Type 2 diabetes mellitus
CRP: C-reactive protein
OGTT: Oral glucose tolerance test
NTD: Neural tube defect
HOMA-IR: Homeostasis model assessment-insulin resistance
HOMA-β: Homeostasis model assessment-β
PCOS: Polycystic ovarian syndrome
IRS: Insulin receptor substrate
